# Mendelian randomization shows causal effects of birth weight and childhood body mass index on the risk of frailty

**DOI:** 10.3389/fpubh.2024.1270698

**Published:** 2024-05-23

**Authors:** Junhao Cui, Shuqin Fu, Lin Zhu, Peng Li, Chunlan Song

**Affiliations:** Children's Hospital Affiliated to Zhengzhou University, Henan Children's Hospital, Zhengzhou Children's Hospital, Zhengzhou, Henan, China

**Keywords:** birth weight, childhood, body mass index, frailty, Mendelian randomization

## Abstract

**Background:**

The association between birth weight and childhood body mass index (BMI) and frailty has been extensively studied, but it is currently unclear whether this relationship is causal.

**Methods:**

We utilized a two-sample Mendelian randomization (MR) methodology to investigate the causal effects of birth weight and childhood BMI on the risk of frailty. Instrumental variables (*p* < 5E-08) strongly associated with own birth weight (*N* = 298,142 infants), offspring birth weight (*N* = 210,267 mothers), and childhood BMI (*N* = 39,620) were identified from large-scale genomic data from genome-wide association studies (GWAS). The frailty status was assessed using the frailty index, which was derived from comprehensive geriatric assessments of older adults within the UK Biobank and the TwinGene database (*N* = 175,226).

**Results:**

Genetically predicted one standard deviation (SD) increase in own birth weight, but not offspring birth weight (maternal-specific), was linked to a decreased frailty index (β per SD increase = −0.068, 95%CI = −0.106 to −0.030, *p* = 3.92E-04). Conversely, genetically predicted one SD increase in childhood BMI was associated with an elevated frailty index (β per SD increase = 0.080, 95%CI = 0.046 to 0.114, *p* = 3.43E-06) with good statistical power (99.8%). The findings remained consistent across sensitivity analyses and showed no horizontal pleiotropy (*p* > 0.05).

**Conclusion:**

This MR study provides evidence supporting a causal relationship between lower birth weight, higher childhood BMI, and an increased risk of frailty.

## Introduction

Frailty is a multifactorial syndrome characterized by decreased physiological reserve and increased vulnerability to adverse health outcomes, such as disability, falls, and mortality in older adults (1, 2). With the aging global population, frailty has become a significant public health issue, leading to substantial economic burdens (3). Therefore, identifying modifiable risk factors is vital for developing preventive approaches against frailty.

The epidemiological studies have found links between early-stage body weight and frailty risk [relative risk ratio (RRR) = 0.40] (4, 5), aligning with the hypothesis of developmental origins of health and disease (DOHaD), which suggested that the fetal and perinatal development could influence disease risk in adulthood (6, 7). Previous studies have demonstrated that a higher birth weight is associated with a reduced risk of frailty (4, 8), whereas, a higher childhood body mass index (BMI) correlates with increased frailty in later life (9), suggesting a relationship between the early-life body metrics and later frailty. For instance, a birth cohort study by Haapanen et al. (5) showed that accelerated BMI gain during childhood is associated with a higher risk of frailty (RRR = 2.36) in men. Due to the susceptibility of observational studies to confounding factors, such as socioeconomic status and environmental exposure (10), it is currently unclear whether the relationship between early-stage body weight and frailty is causal.

Mendelian randomization (MR), utilizing genetic variants as instrumental variables (IV), provides an opportunity to strengthen the causal effect of exposure and corresponding outcomes (11). Since IV is randomly allocated at conception, the MR analysis could mitigate the confounding issues and enhance the robustness of causal inferences. In this MR study, we leveraged an MR approach to investigate the causal impacts of birth weight and childhood BMI on frailty using large-scale genomic data from genome-wide association studies (GWAS).

## Methods and materials

### Data sources

Since birth weight is affected by both infant development (infants) and the intrauterine environment (mothers), it is important to distinguish the role of fetal effect and maternal effect on birth weight (12). Based on the studies published previously (7, 12–16), the birth weight components could be divided into the maternal effect and the fetal effect (16). The data on own birth weight, offspring birth weight (maternal-specific), and childhood BMI were summarized from the Early Growth Genetics (EGG) Consortium, which included 298,142, 210,267, and 39,620 participants respectively, of European descent (17, 18). For the birth weight dataset, the participants with multiple births, birth weights less than 2,500 g or greater than 4,500 g, and gestational age (GA) less than 37 weeks were excluded from the GWAS (17). Furthermore, for the childhood BMI dataset, the BMI was calculated using the formula: weight (kg)/height^2 (m^2^). The summary-level data for childhood BMI were based on a meta-analysis that utilized GWAS datasets from 26 studies, with the mean BMI values ranging from 15.66 to 25.70 kg/m^2^. The data from original studies indicated that the majority of participants (aged between 2 and 10 years) were within the healthy weight range (18). A statistical summary for the frailty index was derived from the latest large-scale GWAS conducted by Atkins et al. (19) (*N* = 175,226). This calculation utilized data from the UK Biobank with 164,610 participants (51.3% females) aged 60–70 years (mean 64.1) and the TwinGene with 10,616 participants (52.5% females) aged 41–87 years (mean 58.3) (19). The frailty index was calculated as the proportion of actual impairments to the total assessed deficits, utilizing 49 and 44 self-reported items related to physical performance, comorbidities, and psychosocial factors for the UK Biobank and TwinGene datasets, respectively, and the details for each item were thoroughly described in the original study (19). The complete statistical summary of GWAS applied in this MR study was from previously published data (Supplementary Figure>Supplementary Table S1) (17–19).

### Selection of instrumental variables

**Figure 1:**
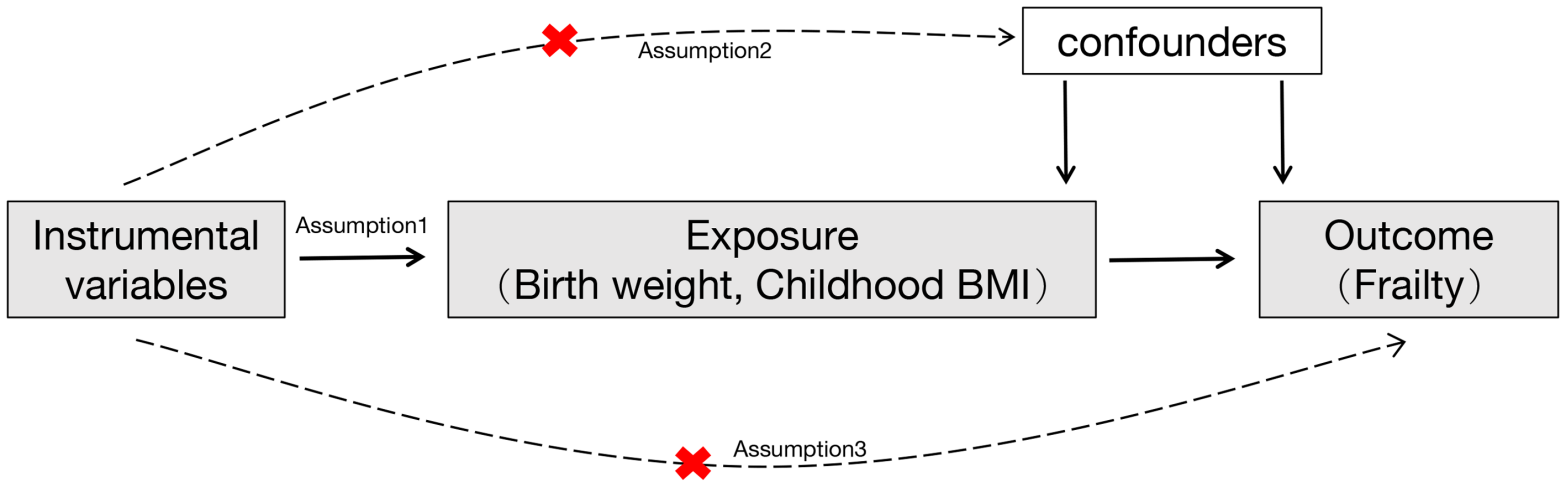
A flowchart of the study design. Red cross indicated that the instrumental variables were not associated with confounders and the outcome.

The selected IV has to satisfy three assumptions (Figure 1): (1) The IV should be associated with the exposures (own birth weight, offspring birth weight, and childhood BMI); (2) The IV should not be associated with confounders; (3) The IV should affect the outcome exclusively through the exposure, without any direct association with the outcome itself (20). To adhere to the outlined assumptions, we selected single nucleotide polymorphisms (SNPs) strongly associated with birth weight and childhood BMI at genome-wide significance levels (*p* < 5E-8). The IV was clumped based on the linkage disequilibrium (LD) structure of European descent (*r*^2^ < 0.001, 10 Mb window). Furthermore, the SNP absent in the GWAS statistical summary on the frailty index will be replaced with a proxy SNP in LD (*r*^2^ = 0.8). To maintain IV validity and to minimize weak instrument bias, an IV with an F-statistic value of less than 10 was removed from the MR analysis (Supplementary Figure>Supplementary Table S2).

### Mendelian randomization analysis

The two-sample MR analysis was conducted using the TwoSampleMR package in R software (21). The inverse variance weighted (IVW) method was applied to assess the causal estimates (β), which represented the change in frailty index per standard deviation (SD) increase in genetic predisposition to the birth weight or the childhood BMI. Furthermore, to evaluate the robustness of the findings, we conducted extensive sensitivity analyses including MR-Egger, weighted median, simple median, maximum likelihood, simple mode, weighted mode, and robust adjusted profile score (RAPS). The MR-Egger intercept test was utilized to examine the potential pleiotropic effects of the IV. In addition, the MR-Pleiotropy RESidual Sum and Outlier (MR-PRESSO) test was used to detect any outliers (22). The statistical power of the MR estimate was assessed using the method previously described^1^ (23). The significance level was set at *p* < 0.05.

## Results

**Figure 2:**
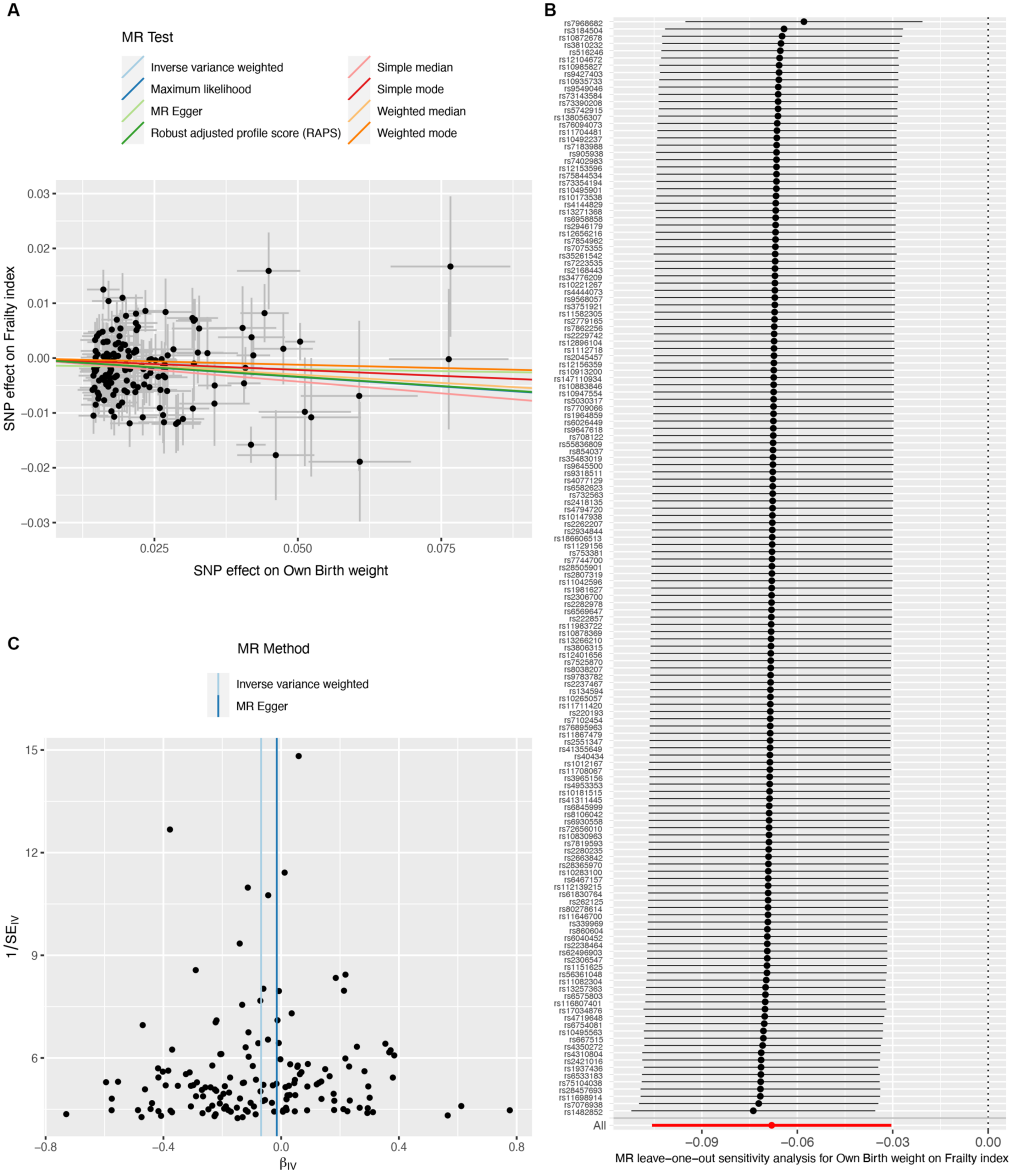
Causal estimates of own birth weight (fetal effect) on frailty index. The scatter plot displayed the causal effects of each single nucleotide polymorphism (SNP) on fetal birth weight and frailty index **(A)**. Leave-one-out plot for the causal relationship between fetal birth weight and frailty index **(B)**. The funnel plot showed the symmetry of the instrumental variables **(C)**. MR, Mendelian randomization.

**Figure 3:**
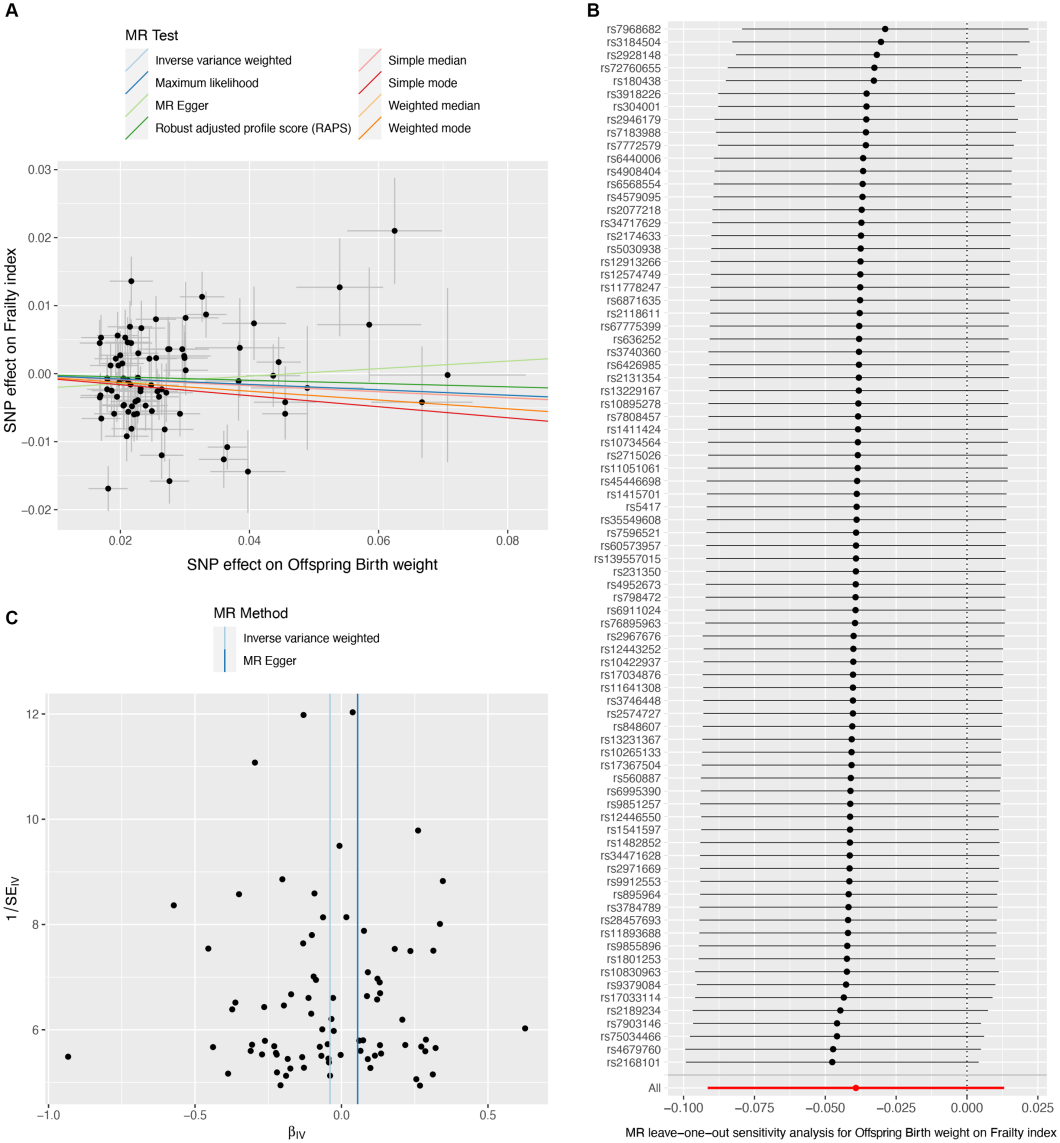
Causal estimates of offspring birth weight (maternal effect) on frailty index. The scatter plot displayed the causal effects of each single nucleotide polymorphism (SNP) on fetal birth weight and frailty index **(A)**. Leave-one-out plot for the causal relationship between fetal birth weight and frailty index **(B)**. The funnel plot showed the symmetry of the instrumental variables **(C)**. MR, Mendelian randomization.

A total of 151 IVs were available for own birth weight, 81 for offspring birth weight, and 16 for childhood BMI. When using fetal own birth weight as the exposure, genetically predicted one SD increase in birth weight was associated with a reduced frailty index (β per SD increase = −0.068, 95%CI = −0.106 to −0.030, *p* = 3.92E-04). The scatter plot (Figure 2A) showed that similar results with the same trend were obtained in the sensitivity analysis (Table 1). The leave-one-out analysis showed that no single SNP was responsible for skewing the estimate (Figure 2B), and the funnel plot revealed no signs of asymmetry (Figure 2C). The Cochran Q test indicated potential heterogeneity, but the MR-Egger intercept test showed no evidence of pleiotropy (Table 2). Three outliers were identified in the MR-PRESSO RSSobs test, but the MR estimate remained significant after removing the outliers from the IV (Table 2). Meanwhile, genetically predicted one SD increase in offspring birth weight (maternal-specific) was also associated with a decreased frailty index (β per SD increase = −0.039, 95%CI = −0.091 to 0.013, *p* = 0.141), but it was not statistically significant (Table 1). The leave-one-out analysis showed that no individual SNP was responsible for the bias of the estimate, and the funnel plot showed no evidence of asymmetry (Figure 3).

**Table 1:** Causal estimates of fetal birth weight and childhood body mass index on frailty.

Exposure	Method	nSNP	Beta^*^	Lower 95%CI	Upper 95%CI	*p*-value
Own birth weight (fetal)	MR Egger	151	−0.015	−0.127	0.097	0.795
	IVW	151	−0.068	−0.106	−0.030	3.92E-04
	Weighted median	151	−0.060	−0.107	−0.013	0.012
	Simple median	151	−0.086	−0.131	−0.040	2.26E-04
	Maximum likelihood	151	−0.069	−0.097	−0.041	1.40E-06
	Simple mode	151	−0.043	−0.183	0.097	0.545
	Weighted mode	151	−0.024	−0.127	0.079	0.646
	RAPS	151	−0.068	−0.106	−0.029	5.50E-04
Offspring birth weight (maternal)	MR Egger	81	0.055	−0.116	0.226	0.528
	IVW	81	−0.039	−0.091	0.013	0.142
	Weighted median	81	−0.044	−0.097	0.010	0.109
	Simple median	81	−0.044	−0.098	0.009	0.106
	Maximum likelihood	81	−0.040	−0.073	−0.006	0.019
	Simple mode	81	−0.081	−0.244	0.082	0.333
	Weighted mode	81	−0.065	−0.208	0.078	0.379
	RAPS	81	−0.024	−0.075	0.027	0.350
Childhood BMI	MR Egger	16	0.093	−0.050	0.237	0.223
	IVW	16	0.080	0.046	0.114	3.43E-06
	Weighted median	16	0.088	0.047	0.129	2.11E-05
	Simple median	16	0.088	0.048	0.129	1.83E-05
	Maximum likelihood	16	0.082	0.053	0.111	3.99E-08
	Simple mode	16	0.093	0.021	0.165	0.022
	Weighted mode	16	0.092	0.030	0.154	0.011
	RAPS	16	0.083	0.052	0.113	1.26E-07

RAPS, robust adjusted profile score; BMI, body mass index; IVW, Inverse variance weighted; SNP, single nucleotide polymorphism; CI, confidence interval. *: The beta-coefficients (causal estimate) represent the change in frailty index per standard deviation increase in corresponding exposures.

**Table 2:** Heterogeneity and pleiotropy test for fetal birth weight and childhood body mass index on frailty index.

Exposure	Cochran Q (P)		Intercept Egger (P)	MR-PRESSO	
	IVW	MR-Egger		Raw	Outlier corrected
Own birth weight (fetal)	280.11 (*p* < 0.001)	278.28 (*p* < 0.001)	−0.001(0.324)	−0.068 (5.23E-04)	−0.064 (6.09E-04)
Offspring birth weight (maternal)	209.35 (*p* < 0.001)	205.98 (*p* < 0.001)	−0.002(0.258)	−0.039 (0.145)	−0.029 (0.193)
Childhood BMI	21.02 (0.136)	20.97 (0.102)	−0.001(0.855)	0.080 (3.18E-04)	NA

BMI, body mass index; IVW, Inverse variance weighted; P, *p* value; MR-PRESSO, Mendelian Randomization Pleiotropy RESidual Sum and Outlier; NA, not applicable.

**Figure 4:**
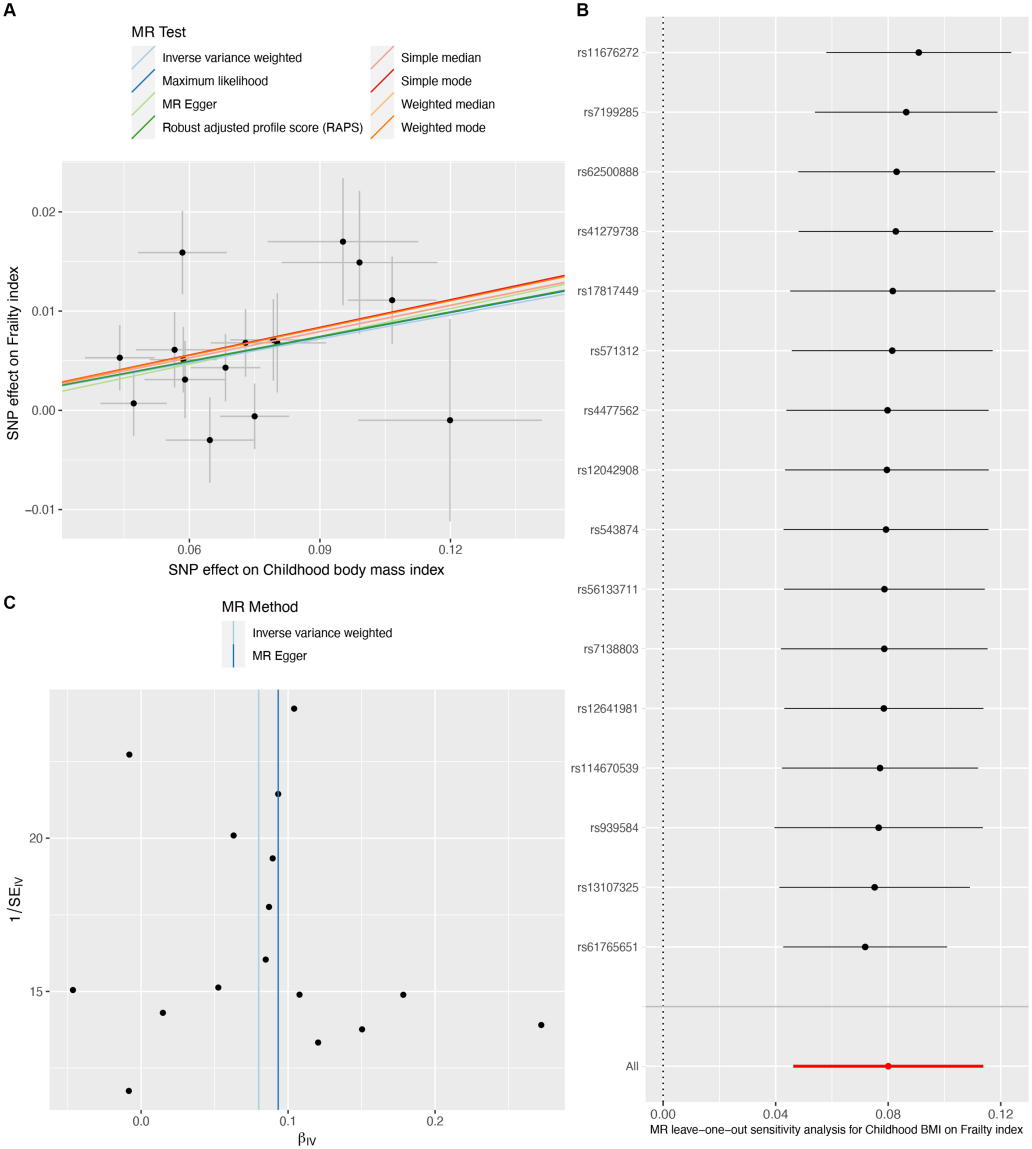
Causal estimates of childhood body mass index on frailty index. The scatter plot showed the causal effects of each single nucleotide polymorphism (SNP) on BMI and frailty index **(A)**. Leave-one-out plot for the causal relationship between childhood BMI and frailty index **(B)**. The funnel plot displayed the symmetry of the instrumental variables **(C)**. MR, Mendelian randomization; BMI, body mass index.

On the contrary, genetically predicted one SD increase in childhood BMI was significantly associated with an increased frailty index (β per SD increase = 0.080, 95%CI = 0.046 to 0.114, *p* = 3.43E-06) (Table 1). The scatter plot (Figure 4A) illustrated that consistent findings with the same trend were observed in the sensitivity analysis (Table 1). The leave-one-out analysis showed that no individual SNP was responsible for biasing the estimate (Figure 4B), and the funnel plot showed no signs of asymmetry (Figure 4C). Heterogeneity and pleiotropy were not observed in the Cochran Q test and MR-Egger intercept test. Furthermore, significant outliers were not detected in the MR-PRESSO test (Table 2). The statistical power for the MR estimates for own birth weight, offspring birth weight, and childhood BMI was 99.8, 65.6, and 99.8% respectively, indicating the robustness of the MR findings.

## Discussion

The present MR study showed that the birth weight and the childhood BMI have a negative and positive association, respectively, with the frailty index. These findings highlight the distinct impact of birth weight and childhood BMI on the risk of frailty in later life, highlighting the importance of strategies that address early-life obesity and promote healthy growth during pregnancy.

Growing evidence has shown that lower birth weight is a critical determinant of long-term health outcomes (5, 24). The MR results of the study confirmed that lower birth weight was causally associated with an increased risk of frailty, which was consistent with the findings from previous observational studies. For instance, the Helsinki birth cohort study led by Haapanen et al. showed a significant association between lower birth weight and higher frailty among community-dwelling older adults (4). Similarly, a cross-sectional analysis of the UK Biobank, including 502,489 individuals aged between 37 and 73 years, showed that people with lower birth weights were more likely to develop frailty later in life (9). The association between lower birth weight and increased risk of frailty might stem from the hypothesis of “fetal programming,” implying that adverse conditions during fetal development could result in permanent changes to bodily systems and organs, thereby escalating the risk of diseases in later life (25). A lower birth weight has been linked to impaired neuroendocrine and immune systems, contributing to the development of frailty in old age (26). Furthermore, based on the understanding that birth weight is influenced by both the development of the fetus and the intrauterine environment, we categorized birth weight factors into fetal and maternal effects as previously described (12, 14, 16, 27). The MR findings indicate that the fetal effect on birth weight has a stronger influence on the risk of frailty than the maternal effect, suggesting that the birth weight-frailty association may be more attributable to the fetal effects than to the maternal effects (intrauterine environment) (16).

Although higher birth weight was linked to a decreased risk of frailty, increasing evidence suggested that individuals with higher BMI might have an increased risk of frailty (28, 29). The MR study confirmed a significant causal relationship between higher childhood BMI and increased risk of frailty in older adults. Data from Sheehan et al.’s study indicates that the risk of developing frailty in the obese population (≥30.00 kg/m^2^) is 4.4 times higher than in the non-obese population (30). A cohort study involving 1,078 individuals demonstrated that accelerated BMI gain in childhood was associated with an increased risk of frailty in men, but not women (5). However, due to the non-availability of gender-specific summary-level data, we were unable to conduct a gender-based MR analysis to further confirm the findings from observational studies. The mechanisms underlying the relationship between childhood BMI and frailty are complex and likely involve multiple factors. Excessive weight gain in childhood can lead to the early development of metabolic and cardiovascular risk factors associated with obesity, such as insulin resistance and chronic inflammation (31, 32), which were implicated in the pathogenesis of frailty, thereby increasing risk in individuals with higher childhood BMI (33, 34). In addition, obesity in childhood can negatively affect musculoskeletal health and physical functioning, potentially exacerbating frailty later in life (35). Furthermore, studies have shown that childhood obesity can lead to adverse changes in bone density and muscle mass (36, 37), which are critical determinants of the risk of frailty. Thus, maintaining a healthy childhood BMI may play a crucial role in reducing the risk of frailty, and strategies such as lifestyle modifications and weight management to prevent childhood obesity may have a beneficial effect on frailty.

Our MR study has several strengths. First, our sensitivity analyses support the main findings and provide evidence that the observed association in MR analysis reflects a consistent trend rather than being the result of analytical bias (Figures 2A, 3A, 4A). Second, the leave-one-out analysis ensures the robustness of our results by showing that no single genetic variant affects the overall MR estimates, thus affirming the validity of our findings (Figures 2B, 3B, 4B). Third, the funnel plot shows the absence of asymmetry (Figures 2C, 3C, 4C), indicating that the causal relationship between birth weight, childhood BMI, and frailty is not likely to be the result of pleiotropy or selection bias (38).

Despite the strengths of the MR design, there are some limitations to consider. First, although every attempt was made to guarantee the relevance and independence of the genetic variables, residual pleiotropy cannot be completely eliminated. Second, not all MR estimates, especially for the MR-Egger method, from the sensitivity analyses are statistically significant (Table 1), which might reduce the robustness of the results. Third, since this study focused on early-stage (infant and childhood) body weight of European descent, the generalizability of the findings to other age populations requires further investigation. Fourth, despite the MR estimates remaining significant after removing outliers, the presence of outliers in the MR-PRESSO test suggests potential biases within IV (Table 2). Fifth, there is potential sample overlap between the datasets for exposure and outcome, which could lead to the overestimation of the effects.

In conclusion, this MR study provides evidence supporting a causal relationship between lower birth weight, higher childhood BMI, and increased frailty index later in life. The findings emphasize that promoting birth weight and preventing childhood obesity may reduce the risk of frailty.

## Data availability statement

The original contributions presented in the study are included in the article/Supplementary Figure>Supplementary material, further inquiries can be directed to the corresponding author.

## Author contributions

JC: Formal analysis, Writing – original draft, Conceptualization, Data curation, Project administration, Supervision, Visualization, Writing – review & editing. SF: Data curation, Validation, Writing – review & editing, Conceptualization, Project administration. LZ: Data curation, Investigation, Writing – review & editing, Conceptualization, Validation. PL: Data curation, Software, Writing – review & editing, Conceptualization. CS: Writing – review & editing, Supervision, Conceptualization.

## Footnotes

^1^https://sb452.shinyapps.io/power/
