# Supplementary material for: Mendelian randomization shows causal effects of birth weight and childhood body mass index on the risk of frailty
Source: Front Public Health. 2024 May 23;12:1270698. doi: 10.3389/fpubh.2024.1270698 (PMC11158621; doi:10.3389/fpubh.2024.1270698)
Supplement: Supplementary file 1 [file Data_Sheet_1.DOCX]

**Supplementary files**

Supplementary Table S1. Summary statistics used in this Mendelian randomization study.

| **Traits** | **Author** | **Year** | **Sample size** | **Population** | **PMID** |
| --- | --- | --- | --- | --- | --- |
| Own birth weight (Fetal) | Warrington et al | 2019 | 298,142 | European | 31043758 |
| Offspring birth weight (Maternal) | Warrington et al | 2019 | 210,267 | European | 31043758 |
| Childhood Body mass index | Vogelezang et al | 2020 | 39,620 | European | 33045005 |
| Frailty index | Atkins et al | 2021 | 175,226 | European | 34431594 |

Supplementary Table S2. Instrumental variables used in this Mendelian randomization study.

| **Exposure** | **SNP** | **Effect Allele** | **Other Allele** | **EAF** | **Beta (exposure)** | **SE (exposure)** | **Pval (exposure)** | **Beta (outcome)** | **SE (outcome)** | **Pval (outcome)** | **F-statistic** |
| --- | --- | --- | --- | --- | --- | --- | --- | --- | --- | --- | --- |
| Own Birthweight (Fetal) | rs1012167 | T | C | 0.599567 | -0.024145 | 0.002661 | 1.2E-19 | 0.0003 | 0.0034 | 0.9263 | 82.33 |
| Own Birthweight (Fetal) | rs10147938 | C | T | 0.613534 | -0.014516 | 0.002643 | 4.04E-08 | 0.0019 | 0.0034 | 0.5709 | 30.16 |
| Own Birthweight (Fetal) | rs10173538 | C | T | 0.628499 | 0.015115 | 0.002665 | 1.45E-08 | -0.0063 | 0.0034 | 0.0628001 | 32.17 |
| Own Birthweight (Fetal) | rs10181515 | C | T | 0.775444 | -0.021433 | 0.003048 | 2.13E-12 | -0.001 | 0.004 | 0.8093 | 49.45 |
| Own Birthweight (Fetal) | rs10221267 | C | T | 0.488394 | -0.016717 | 0.002558 | 6.52E-11 | 0.0045 | 0.0033 | 0.1746 | 42.71 |
| Own Birthweight (Fetal) | rs10265057 | A | G | 0.908483 | -0.027313 | 0.004498 | 1.3E-09 | -0.0005 | 0.0057 | 0.9246 | 36.87 |
| Own Birthweight (Fetal) | rs10283100 | A | G | 0.054129 | -0.041928 | 0.005836 | 7.01E-13 | -0.0038 | 0.0072 | 0.5971 | 51.61 |
| Own Birthweight (Fetal) | rs10492237 | T | C | 0.908849 | 0.026671 | 0.004462 | 2.33E-09 | -0.0117 | 0.0057 | 0.04101 | 35.73 |
| Own Birthweight (Fetal) | rs10495563 | G | A | 0.335993 | -0.022148 | 0.002693 | 2.06E-16 | -0.0057 | 0.0035 | 0.1038 | 67.64 |
| Own Birthweight (Fetal) | rs10495901 | T | G | 0.74358 | -0.016235 | 0.00291 | 2.48E-08 | 0.0077 | 0.0038 | 0.0410696 | 31.13 |
| Own Birthweight (Fetal) | rs10830963 | C | G | 0.722725 | -0.019111 | 0.002868 | 2.79E-11 | -0.0017 | 0.0037 | 0.6423 | 44.40 |
| Own Birthweight (Fetal) | rs10872678 | T | C | 0.723816 | 0.031699 | 0.002848 | 9.78E-29 | -0.0092 | 0.0037 | 0.01287 | 123.88 |
| Own Birthweight (Fetal) | rs10878369 | C | G | 0.736491 | 0.016849 | 0.00294 | 1.03E-08 | -0.0004 | 0.0038 | 0.9135 | 32.84 |
| Own Birthweight (Fetal) | rs10883846 | C | T | 0.614989 | 0.016841 | 0.002618 | 1.3E-10 | -0.0033 | 0.0034 | 0.3282 | 41.38 |
| Own Birthweight (Fetal) | rs10913200 | G | A | 0.972158 | 0.051218 | 0.008047 | 2.02E-10 | -0.0098 | 0.0099 | 0.3223 | 40.51 |
| Own Birthweight (Fetal) | rs10935733 | T | C | 0.399388 | 0.019369 | 0.002641 | 2.33E-13 | -0.0081 | 0.0034 | 0.0163301 | 53.79 |
| Own Birthweight (Fetal) | rs10947554 | C | T | 0.627843 | -0.018452 | 0.002666 | 4.68E-12 | 0.0032 | 0.0034 | 0.3453 | 47.90 |
| Own Birthweight (Fetal) | rs10985827 | T | G | 0.859061 | -0.029972 | 0.003703 | 6.1E-16 | 0.0111 | 0.0048 | 0.0200101 | 65.51 |
| Own Birthweight (Fetal) | rs11042596 | T | G | 0.336246 | 0.026856 | 0.002777 | 4.33E-22 | -0.0019 | 0.0035 | 0.597301 | 93.53 |
| Own Birthweight (Fetal) | rs11082304 | G | T | 0.491864 | -0.015966 | 0.002554 | 4.22E-10 | -0.0048 | 0.0033 | 0.1433 | 39.08 |
| Own Birthweight (Fetal) | rs1112718 | A | G | 0.595901 | -0.025684 | 0.00259 | 3.78E-23 | 0.0034 | 0.0034 | 0.3101 | 98.34 |
| Own Birthweight (Fetal) | rs112139215 | C | A | 0.93248 | -0.047472 | 0.005142 | 2.81E-20 | -0.0017 | 0.0065 | 0.7963 | 85.23 |
| Own Birthweight (Fetal) | rs1129156 | T | C | 0.268561 | 0.017352 | 0.002909 | 2.53E-09 | -0.0018 | 0.0037 | 0.6259 | 35.58 |
| Own Birthweight (Fetal) | rs1151625 | C | T | 0.921525 | 0.026941 | 0.004736 | 1.31E-08 | 0.0084 | 0.0061 | 0.163 | 32.36 |
| Own Birthweight (Fetal) | rs11582305 | A | T | 0.391159 | -0.016589 | 0.002685 | 6.67E-10 | 0.0044 | 0.0034 | 0.1996 | 38.17 |
| Own Birthweight (Fetal) | rs11646700 | A | G | 0.505754 | -0.014661 | 0.002577 | 1.31E-08 | -0.0033 | 0.0033 | 0.3142 | 32.37 |
| Own Birthweight (Fetal) | rs116807401 | T | C | 0.982436 | -0.076602 | 0.010437 | 2.23E-13 | -0.0167 | 0.0128 | 0.1929 | 53.87 |
| Own Birthweight (Fetal) | rs11698914 | C | G | 0.233612 | 0.031861 | 0.003107 | 1.21E-24 | 0.0068 | 0.004 | 0.0887503 | 105.16 |
| Own Birthweight (Fetal) | rs11704481 | G | A | 0.403215 | 0.015216 | 0.002658 | 1.07E-08 | -0.0074 | 0.0034 | 0.0294999 | 32.77 |
| Own Birthweight (Fetal) | rs11708067 | A | G | 0.76245 | -0.040866 | 0.002989 | 1.59E-42 | 0.0018 | 0.0038 | 0.646399 | 186.93 |
| Own Birthweight (Fetal) | rs11711420 | T | G | 0.74671 | 0.018654 | 0.002964 | 3.22E-10 | 0.0004 | 0.0038 | 0.9062 | 39.61 |
| Own Birthweight (Fetal) | rs11867479 | C | T | 0.64661 | -0.017213 | 0.002664 | 1.08E-10 | -0.0005 | 0.0035 | 0.8835 | 41.75 |
| Own Birthweight (Fetal) | rs11983722 | A | T | 0.937628 | 0.031888 | 0.005377 | 3.1E-09 | -0.001 | 0.0068 | 0.8838 | 35.17 |
| Own Birthweight (Fetal) | rs12104672 | T | G | 0.573494 | 0.014383 | 0.002602 | 3.33E-08 | -0.0105 | 0.0033 | 0.00173201 | 30.55 |
| Own Birthweight (Fetal) | rs12153596 | C | T | 0.618925 | 0.01533 | 0.002654 | 7.87E-09 | -0.0068 | 0.0034 | 0.0454904 | 33.36 |
| Own Birthweight (Fetal) | rs12156359 | G | T | 0.951337 | -0.035468 | 0.006132 | 7.48E-09 | 0.0083 | 0.0077 | 0.2824 | 33.46 |
| Own Birthweight (Fetal) | rs12401656 | G | A | 0.865112 | 0.025183 | 0.003795 | 3.36E-11 | -0.0004 | 0.0048 | 0.9365 | 44.03 |
| Own Birthweight (Fetal) | rs12656216 | G | A | 0.211673 | -0.017835 | 0.003163 | 1.75E-08 | 0.0067 | 0.004 | 0.1002 | 31.79 |
| Own Birthweight (Fetal) | rs12896104 | A | G | 0.325609 | -0.01535 | 0.002771 | 3.12E-08 | 0.0041 | 0.0035 | 0.2473 | 30.69 |
| Own Birthweight (Fetal) | rs13257363 | G | A | 0.590761 | 0.01757 | 0.002618 | 2.01E-11 | 0.0052 | 0.0034 | 0.1221 | 45.04 |
| Own Birthweight (Fetal) | rs13266210 | A | G | 0.786195 | 0.0268 | 0.003142 | 1.54E-17 | -0.0012 | 0.0041 | 0.7698 | 72.75 |
| Own Birthweight (Fetal) | rs13271368 | C | T | 0.761298 | 0.020223 | 0.003022 | 2.3E-11 | -0.0062 | 0.0039 | 0.1116 | 44.78 |
| Own Birthweight (Fetal) | rs134594 | C | T | 0.350594 | 0.016799 | 0.002709 | 5.76E-10 | 0.0003 | 0.0035 | 0.9369 | 38.45 |
| Own Birthweight (Fetal) | rs138056307 | A | T | 0.959802 | -0.046157 | 0.006724 | 6.9E-12 | 0.0177 | 0.0082 | 0.0318002 | 47.12 |
| Own Birthweight (Fetal) | rs147110934 | G | T | 0.975416 | 0.05233 | 0.008669 | 1.62E-09 | -0.0108 | 0.0108 | 0.3174 | 36.44 |
| Own Birthweight (Fetal) | rs1482852 | A | G | 0.598574 | 0.050395 | 0.002618 | 1.58E-82 | 0.003 | 0.0034 | 0.3683 | 370.54 |
| Own Birthweight (Fetal) | rs17034876 | C | T | 0.299629 | -0.042243 | 0.002925 | 3.11E-47 | -0.0005 | 0.0037 | 0.9032 | 208.57 |
| Own Birthweight (Fetal) | rs186606513 | G | A | 0.977869 | 0.060699 | 0.010193 | 2.68E-09 | -0.0069 | 0.0137 | 0.6136 | 35.46 |
| Own Birthweight (Fetal) | rs1937436 | G | A | 0.709717 | 0.016095 | 0.002838 | 1.46E-08 | 0.0125 | 0.0036 | 0.000602698 | 32.16 |
| Own Birthweight (Fetal) | rs1964859 | T | C | 0.300161 | 0.015905 | 0.002893 | 3.94E-08 | -0.0036 | 0.0037 | 0.3333 | 30.23 |
| Own Birthweight (Fetal) | rs1981627 | A | G | 0.415155 | -0.017083 | 0.002629 | 8.36E-11 | 0.0012 | 0.0034 | 0.721601 | 42.22 |
| Own Birthweight (Fetal) | rs2045457 | G | A | 0.311648 | 0.016194 | 0.002786 | 6.32E-09 | -0.004 | 0.0036 | 0.2693 | 33.79 |
| Own Birthweight (Fetal) | rs2168443 | T | A | 0.379207 | 0.016595 | 0.00265 | 3.89E-10 | -0.0048 | 0.0034 | 0.1599 | 39.22 |
| Own Birthweight (Fetal) | rs220193 | A | G | 0.225732 | 0.020584 | 0.003116 | 4.07E-11 | 0.0003 | 0.004 | 0.9346 | 43.64 |
| Own Birthweight (Fetal) | rs222857 | C | T | 0.424408 | -0.026478 | 0.002579 | 1.06E-24 | 0.0016 | 0.0033 | 0.630999 | 105.41 |
| Own Birthweight (Fetal) | rs2229742 | G | C | 0.880836 | 0.027215 | 0.004175 | 7.35E-11 | -0.006 | 0.0054 | 0.2728 | 42.49 |
| Own Birthweight (Fetal) | rs2237467 | G | A | 0.779162 | -0.018178 | 0.003112 | 5.34E-09 | -0.0003 | 0.004 | 0.9323 | 34.12 |
| Own Birthweight (Fetal) | rs2238464 | C | T | 0.679415 | -0.015373 | 0.002767 | 2.84E-08 | -0.0045 | 0.0035 | 0.2063 | 30.87 |
| Own Birthweight (Fetal) | rs2262207 | A | G | 0.748032 | 0.01746 | 0.003043 | 9.88E-09 | -0.0021 | 0.0039 | 0.5851 | 32.92 |
| Own Birthweight (Fetal) | rs2280235 | A | G | 0.741043 | -0.018251 | 0.002957 | 6.9E-10 | -0.0025 | 0.0038 | 0.5015 | 38.09 |
| Own Birthweight (Fetal) | rs2282978 | T | C | 0.673948 | -0.018251 | 0.002709 | 1.66E-11 | 0.0011 | 0.0035 | 0.752199 | 45.39 |
| Own Birthweight (Fetal) | rs2306547 | C | T | 0.534191 | 0.018719 | 0.002582 | 4.35E-13 | 0.0028 | 0.0033 | 0.3997 | 52.56 |
| Own Birthweight (Fetal) | rs2306700 | C | T | 0.864261 | -0.022762 | 0.003782 | 1.81E-09 | 0.0014 | 0.0048 | 0.7666 | 36.22 |
| Own Birthweight (Fetal) | rs2418135 | G | A | 0.477735 | -0.019918 | 0.00259 | 1.53E-14 | 0.0022 | 0.0033 | 0.5142 | 59.14 |
| Own Birthweight (Fetal) | rs2421016 | C | T | 0.522263 | -0.020047 | 0.002548 | 3.82E-15 | -0.0077 | 0.0033 | 0.0206001 | 61.90 |
| Own Birthweight (Fetal) | rs2551347 | C | T | 0.250548 | -0.02446 | 0.002971 | 1.93E-16 | 0.0002 | 0.0038 | 0.9573 | 67.78 |
| Own Birthweight (Fetal) | rs262125 | A | T | 0.811221 | 0.01892 | 0.003274 | 7.76E-09 | 0.004 | 0.0042 | 0.3482 | 33.40 |
| Own Birthweight (Fetal) | rs2663842 | G | A | 0.322053 | -0.016122 | 0.002805 | 9.26E-09 | -0.0029 | 0.0036 | 0.4223 | 33.03 |
| Own Birthweight (Fetal) | rs2779165 | G | C | 0.18416 | 0.022108 | 0.003394 | 7.59E-11 | -0.0053 | 0.0043 | 0.2198 | 42.43 |
| Own Birthweight (Fetal) | rs2807319 | A | G | 0.117184 | -0.02307 | 0.004058 | 1.34E-08 | 0.002 | 0.0053 | 0.7059 | 32.32 |
| Own Birthweight (Fetal) | rs28365970 | C | A | 0.74158 | 0.019868 | 0.002948 | 1.65E-11 | 0.0024 | 0.0038 | 0.524499 | 45.42 |
| Own Birthweight (Fetal) | rs28457693 | A | G | 0.890725 | -0.044196 | 0.004212 | 9.89E-26 | -0.0082 | 0.0053 | 0.1226 | 110.10 |
| Own Birthweight (Fetal) | rs28505901 | A | G | 0.248632 | 0.024438 | 0.003085 | 2.46E-15 | -0.0019 | 0.0038 | 0.6269 | 62.75 |
| Own Birthweight (Fetal) | rs2934844 | A | T | 0.32829 | -0.020752 | 0.002816 | 1.8E-13 | 0.002 | 0.0036 | 0.567799 | 54.31 |
| Own Birthweight (Fetal) | rs2946179 | T | C | 0.266507 | -0.019781 | 0.002912 | 1.14E-11 | 0.0059 | 0.0038 | 0.1153 | 46.14 |
| Own Birthweight (Fetal) | rs3184504 | T | C | 0.479244 | -0.022966 | 0.002554 | 2.57E-19 | 0.0108 | 0.0033 | 0.00106699 | 80.86 |
| Own Birthweight (Fetal) | rs339969 | C | A | 0.380652 | -0.016841 | 0.002651 | 2.18E-10 | -0.003 | 0.0034 | 0.3729 | 40.36 |
| Own Birthweight (Fetal) | rs34776209 | C | T | 0.755328 | 0.023223 | 0.002991 | 8.53E-15 | -0.0048 | 0.0038 | 0.2132 | 60.28 |
| Own Birthweight (Fetal) | rs35261542 | C | A | 0.733023 | 0.040621 | 0.002875 | 2.77E-45 | -0.0046 | 0.0037 | 0.2197 | 199.63 |
| Own Birthweight (Fetal) | rs35483019 | C | T | 0.423201 | -0.01462 | 0.002623 | 2.55E-08 | 0.0025 | 0.0033 | 0.4515 | 31.07 |
| Own Birthweight (Fetal) | rs3751921 | A | G | 0.586688 | -0.017474 | 0.002615 | 2.44E-11 | 0.0045 | 0.0034 | 0.1792 | 44.65 |
| Own Birthweight (Fetal) | rs3806315 | G | A | 0.409033 | -0.017678 | 0.002653 | 2.78E-11 | 0.0005 | 0.0034 | 0.8798 | 44.40 |
| Own Birthweight (Fetal) | rs3810232 | A | C | 0.598993 | 0.01799 | 0.00266 | 1.39E-11 | -0.0107 | 0.0034 | 0.001516 | 45.74 |
| Own Birthweight (Fetal) | rs3965156 | A | C | 0.47588 | 0.014749 | 0.002602 | 1.47E-08 | 0.0013 | 0.0033 | 0.6948 | 32.13 |
| Own Birthweight (Fetal) | rs40434 | A | G | 0.609243 | -0.016702 | 0.00265 | 3E-10 | -0.0008 | 0.0034 | 0.8181 | 39.72 |
| Own Birthweight (Fetal) | rs4077129 | T | C | 0.253922 | -0.017276 | 0.003103 | 2.65E-08 | 0.0026 | 0.0038 | 0.4901 | 31.00 |
| Own Birthweight (Fetal) | rs41311445 | A | C | 0.903353 | 0.032564 | 0.004469 | 3.33E-13 | 0.001 | 0.0056 | 0.8548 | 53.09 |
| Own Birthweight (Fetal) | rs41355649 | G | A | 0.934063 | 0.034243 | 0.00531 | 1.17E-10 | 0.0009 | 0.0066 | 0.8971 | 41.59 |
| Own Birthweight (Fetal) | rs4144829 | C | T | 0.267142 | 0.035507 | 0.002915 | 4.32E-34 | -0.005 | 0.0038 | 0.1794 | 148.37 |
| Own Birthweight (Fetal) | rs4310804 | C | G | 0.750767 | 0.023427 | 0.003009 | 7.21E-15 | 0.0086 | 0.0038 | 0.0245901 | 60.62 |
| Own Birthweight (Fetal) | rs4350272 | A | G | 0.269233 | 0.016999 | 0.002879 | 3.64E-09 | 0.0104 | 0.0037 | 0.00540095 | 34.86 |
| Own Birthweight (Fetal) | rs4444073 | A | C | 0.520477 | 0.020182 | 0.002551 | 2.69E-15 | -0.0041 | 0.0033 | 0.2192 | 62.59 |
| Own Birthweight (Fetal) | rs4719648 | T | C | 0.423355 | -0.019079 | 0.002606 | 2.57E-13 | -0.0054 | 0.0034 | 0.1079 | 53.60 |
| Own Birthweight (Fetal) | rs4794720 | G | A | 0.36221 | 0.014846 | 0.002703 | 4.07E-08 | -0.0022 | 0.0035 | 0.5248 | 30.17 |
| Own Birthweight (Fetal) | rs4953353 | G | T | 0.631578 | 0.017903 | 0.0027 | 3.45E-11 | 0.0008 | 0.0034 | 0.8245 | 43.97 |
| Own Birthweight (Fetal) | rs5030317 | C | G | 0.732702 | 0.017273 | 0.002902 | 2.71E-09 | -0.0036 | 0.0037 | 0.3319 | 35.43 |
| Own Birthweight (Fetal) | rs516246 | C | T | 0.506098 | 0.017505 | 0.002566 | 9.34E-12 | -0.0097 | 0.0033 | 0.00343803 | 46.54 |
| Own Birthweight (Fetal) | rs55836809 | A | G | 0.779893 | 0.018354 | 0.003182 | 8.2E-09 | -0.0033 | 0.0041 | 0.4157 | 33.27 |
| Own Birthweight (Fetal) | rs56361048 | T | C | 0.863263 | -0.021757 | 0.003772 | 8.2E-09 | -0.0064 | 0.0048 | 0.1844 | 33.27 |
| Own Birthweight (Fetal) | rs5742915 | T | C | 0.544096 | -0.01476 | 0.002565 | 8.91E-09 | 0.0085 | 0.0033 | 0.0105599 | 33.11 |
| Own Birthweight (Fetal) | rs6026449 | T | C | 0.372918 | -0.016962 | 0.002679 | 2.49E-10 | 0.003 | 0.0034 | 0.3745 | 40.09 |
| Own Birthweight (Fetal) | rs6040452 | G | A | 0.522729 | -0.014996 | 0.002587 | 6.95E-09 | -0.004 | 0.0033 | 0.2266 | 33.60 |
| Own Birthweight (Fetal) | rs61830764 | G | A | 0.623067 | -0.016623 | 0.002727 | 1.12E-09 | -0.003 | 0.0035 | 0.378 | 37.16 |
| Own Birthweight (Fetal) | rs62496903 | C | T | 0.917392 | -0.032797 | 0.004775 | 6.74E-12 | -0.0054 | 0.006 | 0.3667 | 47.18 |
| Own Birthweight (Fetal) | rs6467157 | T | C | 0.712892 | 0.019506 | 0.002887 | 1.47E-11 | 0.0025 | 0.0037 | 0.499401 | 45.65 |
| Own Birthweight (Fetal) | rs6533183 | C | T | 0.352446 | 0.021786 | 0.002696 | 6.78E-16 | 0.0081 | 0.0035 | 0.0191801 | 65.30 |
| Own Birthweight (Fetal) | rs6569647 | T | C | 0.801856 | 0.020035 | 0.003238 | 6.31E-10 | -0.0011 | 0.0042 | 0.7958 | 38.28 |
| Own Birthweight (Fetal) | rs6575803 | C | T | 0.895535 | 0.031625 | 0.004454 | 1.29E-12 | 0.0073 | 0.0055 | 0.1821 | 50.41 |
| Own Birthweight (Fetal) | rs6582623 | T | C | 0.130797 | -0.023591 | 0.003865 | 1.07E-09 | 0.0033 | 0.0049 | 0.5015 | 37.26 |
| Own Birthweight (Fetal) | rs667515 | G | C | 0.61794 | 0.01845 | 0.002704 | 9.32E-12 | 0.007 | 0.0034 | 0.0398199 | 46.56 |
| Own Birthweight (Fetal) | rs6754081 | T | C | 0.156667 | -0.019441 | 0.003557 | 4.71E-08 | -0.011 | 0.0045 | 0.0159599 | 29.87 |
| Own Birthweight (Fetal) | rs6845999 | C | T | 0.56879 | -0.026255 | 0.002565 | 1.5E-24 | 0.0002 | 0.0033 | 0.9623 | 104.77 |
| Own Birthweight (Fetal) | rs6930558 | G | T | 0.252922 | -0.021812 | 0.002996 | 3.44E-13 | -0.0012 | 0.0038 | 0.7457 | 53.00 |
| Own Birthweight (Fetal) | rs6958858 | T | C | 0.513496 | -0.014217 | 0.002602 | 4.76E-08 | 0.0058 | 0.0033 | 0.0782402 | 29.85 |
| Own Birthweight (Fetal) | rs7075355 | G | A | 0.466983 | -0.014565 | 0.002574 | 1.57E-08 | 0.0054 | 0.0033 | 0.1012 | 32.02 |
| Own Birthweight (Fetal) | rs7076938 | C | T | 0.264688 | -0.032051 | 0.002898 | 2.14E-28 | -0.007 | 0.0038 | 0.0624899 | 122.32 |
| Own Birthweight (Fetal) | rs708122 | C | A | 0.68102 | 0.016508 | 0.002765 | 2.45E-09 | -0.0031 | 0.0036 | 0.387 | 35.64 |
| Own Birthweight (Fetal) | rs7102454 | T | C | 0.647495 | -0.015096 | 0.002689 | 2.02E-08 | -0.0007 | 0.0034 | 0.8308 | 31.52 |
| Own Birthweight (Fetal) | rs7183988 | T | G | 0.471182 | -0.01824 | 0.002582 | 1.67E-12 | 0.0059 | 0.0033 | 0.0761202 | 49.90 |
| Own Birthweight (Fetal) | rs7223535 | G | A | 0.732138 | 0.02138 | 0.00291 | 2.12E-13 | -0.005 | 0.0037 | 0.1774 | 53.98 |
| Own Birthweight (Fetal) | rs72656010 | T | C | 0.868303 | 0.028319 | 0.003825 | 1.39E-13 | 0.0016 | 0.0049 | 0.7418 | 54.81 |
| Own Birthweight (Fetal) | rs73143584 | G | A | 0.889782 | -0.028776 | 0.004278 | 1.8E-11 | 0.012 | 0.0053 | 0.0229499 | 45.25 |
| Own Birthweight (Fetal) | rs732563 | T | C | 0.496105 | -0.017423 | 0.002572 | 1.29E-11 | 0.0022 | 0.0033 | 0.5129 | 45.89 |
| Own Birthweight (Fetal) | rs73354194 | T | C | 0.974784 | -0.060799 | 0.008927 | 1.01E-11 | 0.0189 | 0.0109 | 0.0837491 | 46.39 |
| Own Birthweight (Fetal) | rs73390208 | C | T | 0.723553 | 0.018807 | 0.002967 | 2.39E-10 | -0.0087 | 0.0037 | 0.0199499 | 40.18 |
| Own Birthweight (Fetal) | rs7402983 | A | C | 0.405324 | 0.024135 | 0.002685 | 2.63E-19 | -0.0053 | 0.0034 | 0.1212 | 80.80 |
| Own Birthweight (Fetal) | rs75104038 | G | A | 0.940326 | -0.044913 | 0.00552 | 4.28E-16 | -0.0159 | 0.007 | 0.02201 | 66.20 |
| Own Birthweight (Fetal) | rs7525870 | G | A | 0.740264 | 0.016389 | 0.002896 | 1.55E-08 | 0.0001 | 0.0037 | 0.9832 | 32.03 |
| Own Birthweight (Fetal) | rs753381 | T | C | 0.450563 | 0.01512 | 0.002557 | 3.43E-09 | -0.0014 | 0.0033 | 0.6721 | 34.97 |
| Own Birthweight (Fetal) | rs75844534 | C | A | 0.876497 | -0.02593 | 0.003941 | 4.9E-11 | 0.0091 | 0.005 | 0.0710199 | 43.29 |
| Own Birthweight (Fetal) | rs76094073 | C | G | 0.87877 | -0.026513 | 0.003933 | 1.63E-11 | 0.0104 | 0.0051 | 0.0393197 | 45.44 |
| Own Birthweight (Fetal) | rs76895963 | T | G | 0.97881 | -0.076305 | 0.010414 | 2.45E-13 | 0.0002 | 0.0128 | 0.9886 | 53.69 |
| Own Birthweight (Fetal) | rs7709066 | T | C | 0.545329 | -0.014374 | 0.002589 | 2.91E-08 | 0.0032 | 0.0033 | 0.3307 | 30.82 |
| Own Birthweight (Fetal) | rs7744700 | T | A | 0.710579 | 0.01985 | 0.002945 | 1.64E-11 | -0.0017 | 0.0037 | 0.6538 | 45.43 |
| Own Birthweight (Fetal) | rs7819593 | T | C | 0.757083 | -0.02178 | 0.003025 | 6.22E-13 | -0.0015 | 0.0039 | 0.7092 | 51.84 |
| Own Birthweight (Fetal) | rs7854962 | C | G | 0.784812 | 0.021679 | 0.003184 | 1.02E-11 | -0.0062 | 0.0041 | 0.1271 | 46.36 |
| Own Birthweight (Fetal) | rs7862256 | G | A | 0.194735 | -0.021324 | 0.003258 | 6.12E-11 | 0.0048 | 0.0042 | 0.2532 | 42.84 |
| Own Birthweight (Fetal) | rs7968682 | G | T | 0.48633 | 0.041831 | 0.002557 | 4.24E-60 | -0.0158 | 0.0033 | 1.94402E-06 | 267.63 |
| Own Birthweight (Fetal) | rs80278614 | G | A | 0.945468 | -0.040367 | 0.005872 | 6.45E-12 | -0.0055 | 0.0076 | 0.4751 | 47.26 |
| Own Birthweight (Fetal) | rs8038207 | G | T | 0.56945 | 0.01475 | 0.002593 | 1.31E-08 | 0.0001 | 0.0033 | 0.9811 | 32.36 |
| Own Birthweight (Fetal) | rs8106042 | C | G | 0.718496 | -0.020444 | 0.002909 | 2.17E-12 | -0.0013 | 0.0037 | 0.7338 | 49.39 |
| Own Birthweight (Fetal) | rs854037 | A | G | 0.814165 | 0.026507 | 0.003297 | 9.41E-16 | -0.0032 | 0.0042 | 0.4564 | 64.64 |
| Own Birthweight (Fetal) | rs860604 | A | G | 0.156509 | 0.021877 | 0.003524 | 5.52E-10 | 0.0051 | 0.0046 | 0.2695 | 38.54 |
| Own Birthweight (Fetal) | rs905938 | T | C | 0.738356 | -0.026053 | 0.0029 | 2.77E-19 | 0.0058 | 0.0037 | 0.1206 | 80.71 |
| Own Birthweight (Fetal) | rs9318511 | A | C | 0.126856 | -0.026864 | 0.003902 | 5.98E-12 | 0.0035 | 0.0051 | 0.4833 | 47.40 |
| Own Birthweight (Fetal) | rs9427403 | A | C | 0.81463 | 0.020682 | 0.003417 | 1.47E-09 | -0.0119 | 0.0043 | 0.00551506 | 36.63 |
| Own Birthweight (Fetal) | rs9549046 | A | G | 0.117807 | 0.029115 | 0.004062 | 7.97E-13 | -0.0117 | 0.0052 | 0.02537 | 51.37 |
| Own Birthweight (Fetal) | rs9568057 | C | T | 0.735317 | -0.01696 | 0.002938 | 7.98E-09 | 0.0052 | 0.0037 | 0.1634 | 33.32 |
| Own Birthweight (Fetal) | rs9645500 | T | G | 0.305613 | -0.024297 | 0.002768 | 1.75E-18 | 0.0027 | 0.0036 | 0.4485 | 77.05 |
| Own Birthweight (Fetal) | rs9647618 | A | G | 0.784049 | 0.01808 | 0.003171 | 1.22E-08 | -0.0035 | 0.004 | 0.382 | 32.51 |
| Own Birthweight (Fetal) | rs9783782 | T | G | 0.688163 | -0.016125 | 0.00292 | 3.42E-08 | -0.0003 | 0.0036 | 0.925 | 30.50 |
| Offspring Birth weight (Maternal) | rs10265133 | G | T | 0.857555 | 0.027396 | 0.00458 | 2.27E-09 | 0.0036 | 0.0048 | 0.4565 | 35.78 |
| Offspring Birth weight (Maternal) | rs10422937 | T | C | 0.483874 | 0.019698 | 0.003134 | 3.38E-10 | 0.0012 | 0.0034 | 0.7184 | 39.50 |
| Offspring Birth weight (Maternal) | rs10734564 | A | G | 0.17922 | 0.027112 | 0.004103 | 4.01E-11 | -0.0028 | 0.0043 | 0.5108 | 43.66 |
| Offspring Birth weight (Maternal) | rs10830963 | C | G | 0.721234 | -0.044519 | 0.003417 | 9.06E-39 | -0.0017 | 0.0037 | 0.6423 | 169.74 |
| Offspring Birth weight (Maternal) | rs10895278 | T | C | 0.659753 | -0.023117 | 0.003215 | 6.73E-13 | 0.0026 | 0.0035 | 0.4587 | 51.70 |
| Offspring Birth weight (Maternal) | rs11051061 | G | A | 0.732045 | -0.026403 | 0.003464 | 2.59E-14 | 0.0023 | 0.0038 | 0.544 | 58.10 |
| Offspring Birth weight (Maternal) | rs11641308 | T | C | 0.346035 | 0.020298 | 0.00333 | 1.12E-09 | 0.0015 | 0.0035 | 0.6673 | 37.15 |
| Offspring Birth weight (Maternal) | rs11778247 | G | A | 0.834343 | 0.024878 | 0.004215 | 3.68E-09 | -0.0055 | 0.0045 | 0.2155 | 34.84 |
| Offspring Birth weight (Maternal) | rs11893688 | C | T | 0.33397 | -0.019572 | 0.003212 | 1.13E-09 | -0.0056 | 0.0035 | 0.1089 | 37.13 |
| Offspring Birth weight (Maternal) | rs12443252 | C | T | 0.450332 | -0.018479 | 0.003154 | 4.8E-09 | -0.0012 | 0.0033 | 0.7096 | 34.33 |
| Offspring Birth weight (Maternal) | rs12446550 | G | A | 0.580966 | -0.016794 | 0.003073 | 4.74E-08 | -0.0045 | 0.0034 | 0.1807 | 29.87 |
| Offspring Birth weight (Maternal) | rs12574749 | C | A | 0.719504 | 0.020571 | 0.00339 | 1.33E-09 | -0.0046 | 0.0037 | 0.2163 | 36.82 |
| Offspring Birth weight (Maternal) | rs12913266 | G | A | 0.608861 | 0.022692 | 0.00311 | 3.1E-13 | -0.0039 | 0.0034 | 0.2522 | 53.24 |
| Offspring Birth weight (Maternal) | rs13229167 | G | A | 0.725981 | 0.019475 | 0.00342 | 1.27E-08 | -0.0034 | 0.0037 | 0.3674 | 32.43 |
| Offspring Birth weight (Maternal) | rs13231367 | G | A | 0.704766 | 0.019973 | 0.003325 | 1.95E-09 | 0.0027 | 0.0036 | 0.453 | 36.08 |
| Offspring Birth weight (Maternal) | rs139557015 | T | C | 0.964846 | 0.048959 | 0.008723 | 2.04E-08 | -0.0021 | 0.0091 | 0.816 | 31.50 |
| Offspring Birth weight (Maternal) | rs1411424 | G | A | 0.477491 | -0.023137 | 0.00304 | 2.87E-14 | 0.0022 | 0.0033 | 0.5131 | 57.92 |
| Offspring Birth weight (Maternal) | rs1415701 | G | A | 0.729476 | 0.021569 | 0.003489 | 6.54E-10 | -0.0016 | 0.0038 | 0.6653 | 38.22 |
| Offspring Birth weight (Maternal) | rs1482852 | A | G | 0.598589 | 0.022763 | 0.003103 | 2.3E-13 | 0.003 | 0.0034 | 0.3683 | 53.81 |
| Offspring Birth weight (Maternal) | rs1541597 | G | T | 0.795187 | -0.020749 | 0.003784 | 4.26E-08 | -0.0053 | 0.0041 | 0.1995 | 30.07 |
| Offspring Birth weight (Maternal) | rs17033114 | T | C | 0.933851 | 0.053973 | 0.006726 | 1.07E-15 | 0.0127 | 0.0072 | 0.07894 | 64.39 |
| Offspring Birth weight (Maternal) | rs17034876 | C | T | 0.303917 | -0.030115 | 0.00342 | 1.36E-18 | -0.0005 | 0.0037 | 0.9032 | 77.54 |
| Offspring Birth weight (Maternal) | rs17367504 | A | G | 0.833326 | -0.029878 | 0.004098 | 3.21E-13 | -0.0026 | 0.0045 | 0.5546 | 53.16 |
| Offspring Birth weight (Maternal) | rs1801253 | G | C | 0.265816 | -0.021484 | 0.003457 | 5.28E-10 | -0.0069 | 0.0038 | 0.06939 | 38.62 |
| Offspring Birth weight (Maternal) | rs180438 | G | A | 0.194904 | 0.036016 | 0.00385 | 8.85E-21 | -0.0126 | 0.0042 | 0.002732 | 87.51 |
| Offspring Birth weight (Maternal) | rs2077218 | G | A | 0.242495 | 0.02258 | 0.003562 | 2.37E-10 | -0.0059 | 0.0039 | 0.1318 | 40.18 |
| Offspring Birth weight (Maternal) | rs2118611 | T | C | 0.798288 | 0.021797 | 0.00385 | 1.54E-08 | -0.0048 | 0.0042 | 0.2576 | 32.05 |
| Offspring Birth weight (Maternal) | rs2131354 | G | A | 0.473209 | -0.025738 | 0.003144 | 2.82E-16 | 0.0026 | 0.0033 | 0.4351 | 67.02 |
| Offspring Birth weight (Maternal) | rs2168101 | C | A | 0.691309 | 0.032655 | 0.003447 | 2.92E-21 | 0.0113 | 0.0037 | 0.002207 | 89.75 |
| Offspring Birth weight (Maternal) | rs2174633 | A | C | 0.267868 | 0.023908 | 0.00344 | 3.78E-12 | -0.0047 | 0.0037 | 0.2054 | 48.30 |
| Offspring Birth weight (Maternal) | rs2189234 | T | G | 0.384238 | -0.025509 | 0.003116 | 2.86E-16 | -0.008 | 0.0034 | 0.01903 | 67.02 |
| Offspring Birth weight (Maternal) | rs231350 | C | A | 0.645376 | 0.017947 | 0.003286 | 4.83E-08 | -0.0007 | 0.0035 | 0.8291 | 29.83 |
| Offspring Birth weight (Maternal) | rs2574727 | G | A | 0.946073 | 0.038502 | 0.006943 | 0.00000003 | 0.0038 | 0.0073 | 0.6 | 30.75 |
| Offspring Birth weight (Maternal) | rs2715026 | G | C | 0.597888 | 0.017949 | 0.00312 | 8.97E-09 | -0.0023 | 0.0034 | 0.4983 | 33.10 |
| Offspring Birth weight (Maternal) | rs28457693 | A | G | 0.893729 | -0.030104 | 0.0051 | 3.69E-09 | -0.0082 | 0.0053 | 0.1226 | 34.84 |
| Offspring Birth weight (Maternal) | rs2928148 | G | A | 0.477229 | -0.018111 | 0.003038 | 2.57E-09 | 0.0169 | 0.0033 | 3.629E-07 | 35.54 |
| Offspring Birth weight (Maternal) | rs2946179 | T | C | 0.26055 | -0.045528 | 0.003556 | 1.76E-37 | 0.0059 | 0.0038 | 0.1153 | 163.92 |
| Offspring Birth weight (Maternal) | rs2967676 | A | C | 0.841635 | 0.043676 | 0.004191 | 2.15E-25 | -0.0003 | 0.0046 | 0.9523 | 108.60 |
| Offspring Birth weight (Maternal) | rs2971669 | C | T | 0.781285 | -0.027606 | 0.003694 | 8.18E-14 | -0.0036 | 0.004 | 0.3743 | 55.85 |
| Offspring Birth weight (Maternal) | rs304001 | G | A | 0.394362 | 0.021718 | 0.003101 | 2.59E-12 | -0.0081 | 0.0034 | 0.01715 | 49.05 |
| Offspring Birth weight (Maternal) | rs3184504 | T | C | 0.482144 | -0.036552 | 0.003024 | 1.37E-33 | 0.0108 | 0.0033 | 0.001067 | 146.10 |
| Offspring Birth weight (Maternal) | rs34471628 | A | G | 0.960747 | 0.058548 | 0.008049 | 3.65E-13 | 0.0072 | 0.0084 | 0.3893 | 52.91 |
| Offspring Birth weight (Maternal) | rs34717629 | G | A | 0.785645 | -0.022126 | 0.003827 | 7.59E-09 | 0.006 | 0.004 | 0.1377 | 33.43 |
| Offspring Birth weight (Maternal) | rs35549608 | T | C | 0.839555 | -0.02477 | 0.004276 | 7.08E-09 | 0.0017 | 0.0045 | 0.6995 | 33.56 |
| Offspring Birth weight (Maternal) | rs3740360 | A | C | 0.88569 | -0.045529 | 0.004841 | 5.51E-21 | 0.0042 | 0.0053 | 0.4309 | 88.45 |
| Offspring Birth weight (Maternal) | rs3746448 | C | T | 0.83551 | 0.024491 | 0.004226 | 0.000000007 | 0.0022 | 0.0045 | 0.6202 | 33.59 |
| Offspring Birth weight (Maternal) | rs3784789 | C | G | 0.325645 | -0.021671 | 0.003236 | 2.2E-11 | -0.0045 | 0.0035 | 0.2051 | 44.85 |
| Offspring Birth weight (Maternal) | rs3918226 | C | T | 0.918956 | 0.039755 | 0.005823 | 8.99E-12 | -0.0144 | 0.0061 | 0.01896 | 46.61 |
| Offspring Birth weight (Maternal) | rs45446698 | T | G | 0.958189 | -0.066721 | 0.007832 | 1.69E-17 | 0.0042 | 0.0082 | 0.6139 | 72.57 |
| Offspring Birth weight (Maternal) | rs4579095 | A | G | 0.593347 | -0.019033 | 0.003099 | 8.46E-10 | 0.0059 | 0.0034 | 0.08038 | 37.72 |
| Offspring Birth weight (Maternal) | rs4679760 | C | G | 0.413838 | -0.033272 | 0.003188 | 1.83E-25 | -0.0087 | 0.0034 | 0.009908 | 108.92 |
| Offspring Birth weight (Maternal) | rs4908404 | T | C | 0.530117 | -0.01705 | 0.003064 | 0.000000027 | 0.0066 | 0.0033 | 0.04595 | 30.96 |
| Offspring Birth weight (Maternal) | rs4952673 | G | A | 0.52582 | -0.020476 | 0.003051 | 2.01E-11 | 0.0007 | 0.0033 | 0.8398 | 45.04 |
| Offspring Birth weight (Maternal) | rs5030938 | C | T | 0.312963 | -0.020475 | 0.003282 | 4.56E-10 | 0.0047 | 0.0036 | 0.1935 | 38.92 |
| Offspring Birth weight (Maternal) | rs5417 | C | A | 0.427971 | -0.019827 | 0.003074 | 1.16E-10 | 0.0013 | 0.0033 | 0.7016 | 41.60 |
| Offspring Birth weight (Maternal) | rs560887 | T | C | 0.299167 | -0.025535 | 0.003307 | 1.2E-14 | -0.0023 | 0.0036 | 0.5206 | 59.62 |
| Offspring Birth weight (Maternal) | rs60573957 | C | T | 0.492777 | -0.017993 | 0.003138 | 0.00000001 | 0.0008 | 0.0033 | 0.8109 | 32.88 |
| Offspring Birth weight (Maternal) | rs636252 | T | C | 0.407676 | 0.016815 | 0.003077 | 4.73E-08 | -0.0035 | 0.0034 | 0.2982 | 29.86 |
| Offspring Birth weight (Maternal) | rs6426985 | G | A | 0.557776 | -0.016914 | 0.003063 | 3.45E-08 | 0.0032 | 0.0033 | 0.3302 | 30.49 |
| Offspring Birth weight (Maternal) | rs6440006 | G | A | 0.552277 | -0.021224 | 0.003061 | 4.27E-12 | 0.0056 | 0.0033 | 0.09527 | 48.08 |
| Offspring Birth weight (Maternal) | rs6568554 | C | A | 0.856092 | -0.026865 | 0.004455 | 1.69E-09 | 0.0082 | 0.0047 | 0.07869 | 36.36 |
| Offspring Birth weight (Maternal) | rs67775399 | T | C | 0.773401 | -0.022328 | 0.003869 | 8.06E-09 | 0.0041 | 0.0041 | 0.3156 | 33.30 |
| Offspring Birth weight (Maternal) | rs6871635 | A | G | 0.436834 | -0.025981 | 0.003078 | 3.31E-17 | 0.0034 | 0.0034 | 0.3111 | 71.25 |
| Offspring Birth weight (Maternal) | rs6911024 | T | C | 0.90242 | 0.038308 | 0.005206 | 1.93E-13 | -0.0011 | 0.0058 | 0.855 | 54.15 |
| Offspring Birth weight (Maternal) | rs6995390 | A | T | 0.835259 | -0.029588 | 0.004132 | 8.36E-13 | -0.0036 | 0.0045 | 0.4206 | 51.28 |
| Offspring Birth weight (Maternal) | rs7183988 | T | G | 0.47404 | -0.029236 | 0.003141 | 1.37E-20 | 0.0059 | 0.0033 | 0.07612 | 86.64 |
| Offspring Birth weight (Maternal) | rs72760655 | C | A | 0.67813 | 0.026397 | 0.00336 | 4.12E-15 | -0.012 | 0.0035 | 0.0007415 | 61.72 |
| Offspring Birth weight (Maternal) | rs75034466 | C | T | 0.952161 | -0.062484 | 0.007329 | 1.6E-17 | -0.021 | 0.0078 | 0.007033 | 72.68 |
| Offspring Birth weight (Maternal) | rs7596521 | A | G | 0.728631 | -0.021185 | 0.003413 | 5.53E-10 | 0.001 | 0.0037 | 0.7947 | 38.53 |
| Offspring Birth weight (Maternal) | rs76895963 | T | G | 0.979146 | -0.070688 | 0.012095 | 5.22E-09 | 0.0002 | 0.0128 | 0.9886 | 34.16 |
| Offspring Birth weight (Maternal) | rs7772579 | A | C | 0.718456 | 0.020981 | 0.003381 | 5.61E-10 | -0.0092 | 0.0037 | 0.01231 | 38.51 |
| Offspring Birth weight (Maternal) | rs7808457 | T | A | 0.414271 | -0.018634 | 0.003081 | 1.51E-09 | 0.0025 | 0.0034 | 0.4577 | 36.58 |
| Offspring Birth weight (Maternal) | rs7903146 | C | T | 0.708772 | -0.021695 | 0.003345 | 9.08E-11 | -0.0136 | 0.0036 | 0.0001975 | 42.07 |
| Offspring Birth weight (Maternal) | rs7968682 | G | T | 0.485956 | 0.027601 | 0.003047 | 1.41E-19 | -0.0158 | 0.0033 | 0.000001944 | 82.05 |
| Offspring Birth weight (Maternal) | rs798472 | T | C | 0.739748 | 0.022716 | 0.003617 | 3.48E-10 | -0.0006 | 0.0038 | 0.8803 | 39.44 |
| Offspring Birth weight (Maternal) | rs848607 | G | A | 0.684638 | 0.01928 | 0.003268 | 3.72E-09 | 0.0022 | 0.0035 | 0.5298 | 34.81 |
| Offspring Birth weight (Maternal) | rs895964 | G | A | 0.526454 | 0.017001 | 0.003056 | 2.71E-08 | 0.0053 | 0.0033 | 0.1087 | 30.95 |
| Offspring Birth weight (Maternal) | rs9379084 | G | A | 0.883436 | 0.040696 | 0.004916 | 1.32E-16 | 0.0074 | 0.0054 | 0.1696 | 68.53 |
| Offspring Birth weight (Maternal) | rs9851257 | T | A | 0.743524 | 0.029942 | 0.003586 | 7.18E-17 | 0.0023 | 0.0038 | 0.5503 | 69.72 |
| Offspring Birth weight (Maternal) | rs9855896 | A | G | 0.778414 | -0.023247 | 0.003668 | 2.41E-10 | -0.0067 | 0.004 | 0.0945 | 40.17 |
| Offspring Birth weight (Maternal) | rs9912553 | C | G | 0.275032 | -0.021129 | 0.003508 | 1.76E-09 | -0.0046 | 0.0037 | 0.2146 | 36.28 |
| Childhood BMI | rs114670539 | T | C | NA | 0.0991 | 0.0179 | 3.15697E-08 | 0.0149 | 0.0072 | 0.0395203 | 30.65 |
| Childhood BMI | rs11676272 | G | A | NA | 0.075 | 0.0079 | 2.37301E-21 | -0.0006 | 0.0033 | 0.8577 | 90.13 |
| Childhood BMI | rs12042908 | G | A | NA | -0.0586 | 0.0077 | 2.76503E-14 | -0.0051 | 0.0033 | 0.1253 | 57.92 |
| Childhood BMI | rs12641981 | T | C | NA | 0.044 | 0.008 | 4.18697E-08 | 0.0053 | 0.0033 | 0.115 | 30.25 |
| Childhood BMI | rs13107325 | T | C | NA | 0.0953 | 0.0173 | 3.51301E-08 | 0.017 | 0.0064 | 0.00794694 | 30.34 |
| Childhood BMI | rs17817449 | G | T | NA | 0.0683 | 0.008 | 1.69005E-17 | 0.0043 | 0.0034 | 0.2056 | 72.89 |
| Childhood BMI | rs41279738 | G | T | NA | 0.1199 | 0.0211 | 1.29601E-08 | -0.001 | 0.0102 | 0.9192 | 32.29 |
| Childhood BMI | rs4477562 | T | C | NA | 0.0802 | 0.0112 | 8.28705E-13 | 0.0068 | 0.005 | 0.1713 | 51.27 |
| Childhood BMI | rs543874 | G | A | NA | 0.0793 | 0.0099 | 1.61585E-15 | 0.0071 | 0.0041 | 0.0822205 | 64.16 |
| Childhood BMI | rs56133711 | A | G | NA | 0.0566 | 0.0089 | 2.00101E-10 | 0.0061 | 0.0038 | 0.1055 | 40.44 |
| Childhood BMI | rs571312 | A | C | NA | 0.059 | 0.0093 | 1.996E-10 | 0.0031 | 0.0039 | 0.4213 | 40.25 |
| Childhood BMI | rs61765651 | T | C | NA | -0.0584 | 0.0102 | 9.50298E-09 | -0.0159 | 0.0042 | 0.000149699 | 32.78 |
| Childhood BMI | rs62500888 | G | A | NA | -0.0472 | 0.0076 | 6.91003E-10 | -0.0007 | 0.0033 | 0.8243 | 38.57 |
| Childhood BMI | rs7138803 | A | G | NA | 0.0729 | 0.008 | 7.11541E-20 | 0.0068 | 0.0034 | 0.0451004 | 83.03 |
| Childhood BMI | rs7199285 | T | C | NA | -0.0647 | 0.0101 | 1.33999E-10 | 0.003 | 0.0043 | 0.4822 | 41.03 |
| Childhood BMI | rs939584 | T | C | NA | 0.1066 | 0.0102 | 8.85116E-26 | 0.0111 | 0.0044 | 0.01065 | 109.22 |

BMI, body mass index; SNP, single nucleotide polymorphism; EAF, effect allele frequency; NA, not applicable.
